# Stress fractures in older athletes: a case report and literature review

**DOI:** 10.1002/ccr3.954

**Published:** 2017-04-18

**Authors:** André Barros, Samir Karmali, Bárbara Rosa, Ricardo Gonçalves

**Affiliations:** ^1^ Hospital de Vila Franca de XiraVila Franca de XiraLisboa2600‐009Portugal; ^2^ Orthopaedic SurgeryHospital Privado de Gaia^⍛^Rua Fernão de Magalhães, n^⍛^ 2, Fração EVila Nova de Gaia4404‐501Portugal

**Keywords:** Athletes, fracture, older, stress

## Abstract

The incidence of stress injuries in older athletes is increasing, associated with a more active older population. The same principles apply for its prevention and treatment, but older athletes usually present a more adverse outcome. It is mandatory to raise awareness to this common, but frequently neglected pathology.

## Introduction

Stress fractures are caused by an imbalance between bone reabsorption and bone formation. This occurs due to a dominance of osteoclast activity over osteoblast activity 1, usually because of high intensity training with submaximal loads applied to the bone without due rest 2. With continued stress, microfractures occur and may after enlarge into an actual cortical break, or stress fracture 1. They commonly occur in young active people, such as athletes or military recruits 3, and are responsible for 0.7–20% of all sports medicine clinic injuries 4. They represent approximately 10% of all overuse lesions in sport practice and are particularly common in track and field athletes (documented annual occurrences of up to 20% in runners) 4, 5, 6. Almost all publications of stress fractures report in the range of 10–30 years of age, with studies in older athletes being scarce 7, 8, 9. With the increased sports participation in the older population, the incidence of these fractures is becoming more relevant, and more studies on this topic needed.

## Case Report

**Figure 1:**
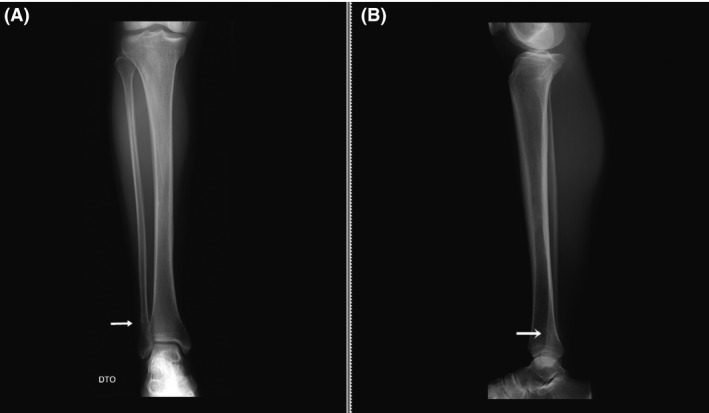
Right leg radiography, AP (A) and lateral (B) incidences, showing fibular fracture, with hypertrophic callus (arrow).

**Figure 2:**
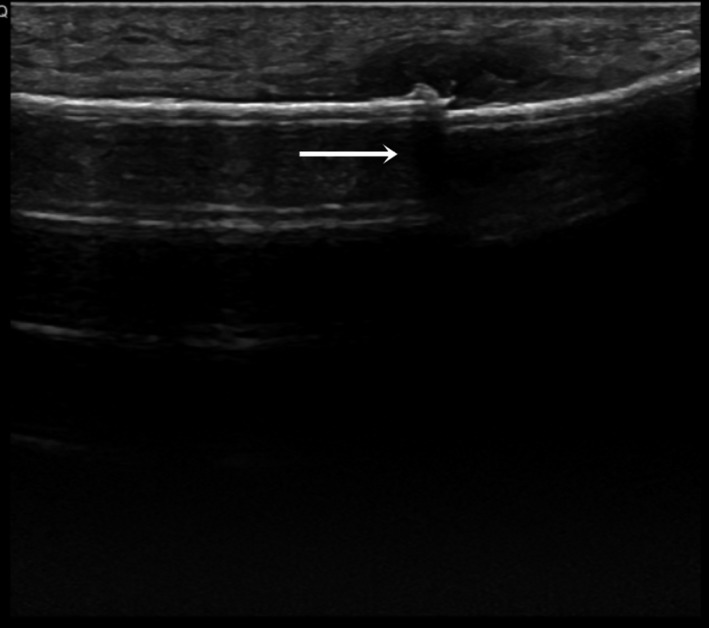
Right leg ultrasound demonstrating fibular cortical discontinuity and soft tissue edema (arrow).

**Figure 3:**
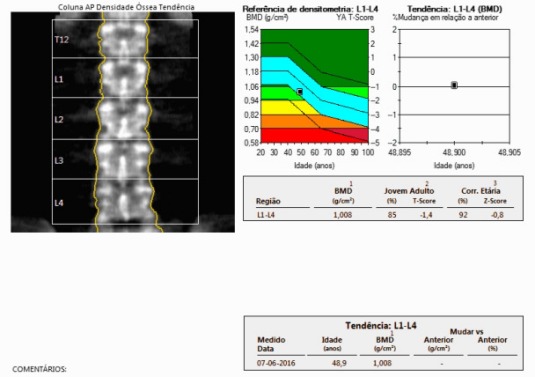
Bone densitometry.

We report a clinical case of a 48‐year‐old female runner with complaints of mechanical pain in the right calcaneal and pre‐tibial regions, lasting for about 7 weeks and without any traumatic episode. The patient was trying to manage the pain with self‐medicated analgesics and non‐medical prescribed physiotherapy. Clinical examination revealed slight lateral‐side edema, without any other signs of inflammation. There was pain at the distal peroneus and extensor retinaculum, upon palpation. Radiography documented an aligned, transverse, distal third, fibular stress fracture with evident bone callus (Fig. 1). Ultrasonography confirmed the existence of a fibular fracture and excluded lesions in the adjacent soft tissues (Fig. 2). Upon exploring the previous medical history, the patient reported irregularities in her menstrual cycle in relation to a hormonal imbalance, which was already being addressed by a rheumatology assistant. The patient was discharged home and maintained a non‐weight bearing regimen for the right limb for 2 weeks. Hormonal tests revealed the following: TSH 0.473 mUI/L (0.550–4.780 mUI/L), T3 0.87 ng/mL (0.60–1.81 ng/mL), free T3 2.79 pg/mL (2.3–4.2 pg/mL), T4 9.2 *μ*g/dL (4.5–10.9 *μ*g/dL), free T4 1.15 ng/dL (0.89–1.76 ng/dL), FSH 6.9 UI/L, progesterone 0.71 ng/mL, ACTH 14.6 pg/mL (<46.0 pg/mL); bone densitometry (Fig. 3): L2L4 T‐score −1.4 (osteopenic), femoral neck T‐score ‐0.8 (normal). At twelve weeks post‐injury, the patient was, against medical opinion, running 7 km a day and reporting only light pain. Radiographic evaluation showed a hypertrophic callus, with no misalignment.

**Figure 4:**
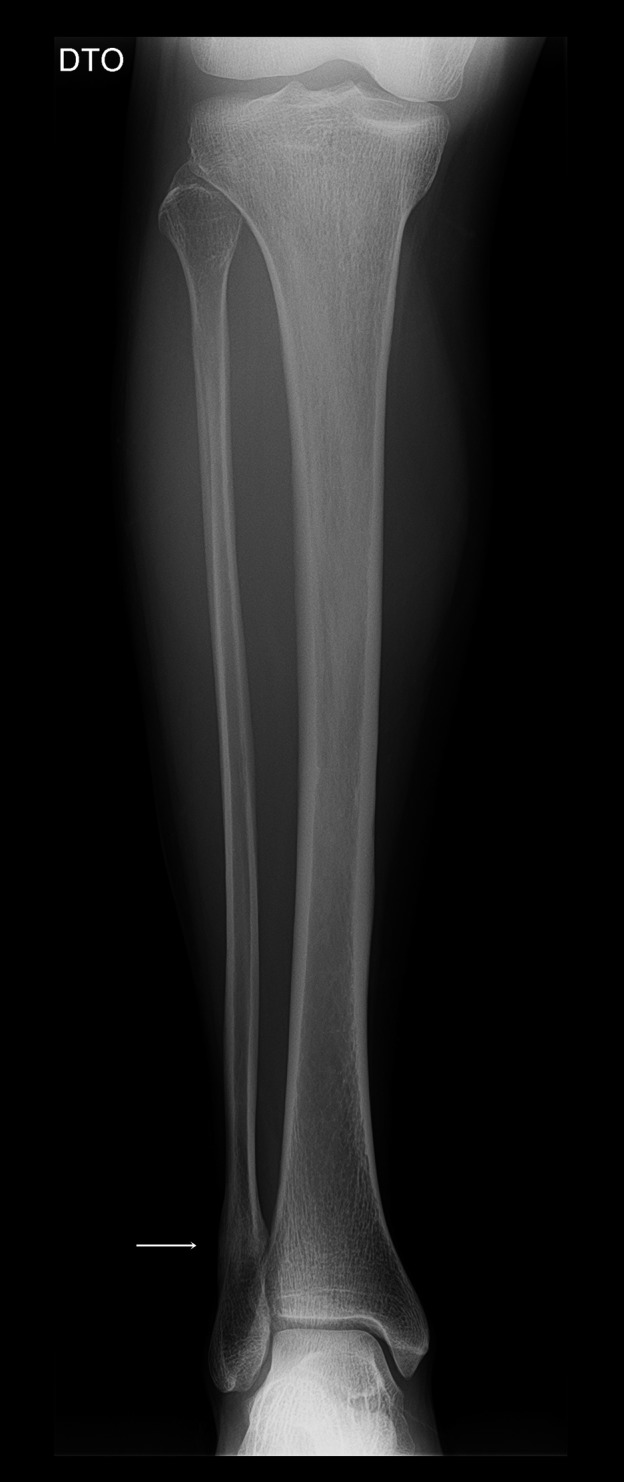
Right leg radiography, AP view, at six‐month follow‐up, confirming fracture consolidation with callus and bony remodeling (arrow).

Follow‐up at 6 months indicated a pain free athlete, capable of performing base training without symptoms. Radiography documented callus reabsorption and bone remodeling, with fracture consolidation (Fig. 4).

## Discussion

### Pathophysiology and associated risk factors

Almost every bone is susceptible to a stress fracture, but 95% are reported to occur in the lower extremities 10. In runners, stress fractures usually occur in the cortical bone 7. Generally, stress fractures affect the diaphysis of long bones or the shell of square bones due to the slower turnover rate of these regions 8. In decreasing order of incidence, typical locations of fatigue fractures include the following: tibia (33%), tarsal bones (20%), metatarsals (20%), femur (11%), fibula (7%), and pelvis (7%) 4, 9. These injuries are frequently encountered across all patient groups, regardless of age or sex 7. According to most studies, there is a higher incidence of stress fractures in women than men 10. Although the origin of this disparity is probably multifactorial, it has been frequently reported to be related to the so‐called female athlete triad 1. This consists of a conjugation of low‐energy availability (with or without eating disorders), menstrual dysfunction, and low bone mineral density, consequently leading to a high propensity for stress fractures by an interplay of nutritional deficits, hypothalamic and estrogen abnormalities 11, 12. In support of this theory, several studies report that stress fractures are more common in amenorrheic or oligomenorrheic women 5, 12, 13.

Matilla et al. 14. reported that older ages are associated with a higher incidence of bone stress injuries (HR 2.1; 95% CI: 1.4–3.1), with poor muscle strength and a poor result on a 12‐min run seeming to be significant risk factors. Breer et al. 15. reported that the same risk factors apply for stress factors in the young and older populations. It seems that the incidence of these injuries has a multifactorial basis and is not exclusively related to lower bone mineral density (the mean t‐score is reported in the osteopenic range and not for osteoporosis). Vitamin D insufficiency is equally related to stress fractures in young and older people, and its supplementation has proven successful in reducing stress fractures in the younger population 16, although its efficacy is yet to be proven in older patients 17. In addition to vitamin D insufficiency, calcium metabolism may also be affected by the frequent use of proton‐pump inhibitors among the elderly. Proton‐pump inhibitor use has previously been associated with proximal femur fractures 18, but the supposed relation with stress fractures has not been reported. Eating disorders, such as anorexia nervosa, have been associated with stress fractures in younger adults 6, but these conditions are considerably less frequent in the elderly. With respect to fracture location, there are also differences between the age groups – metatarsal fractures are the most frequent in older patients, whereas tibial lesions are the most frequent stress fractures in the young.

Regarding stress fractures in the elderly, it is postulated that these injuries might be related to the poor adaptation of the bony microarchitecture to imposed physical stress, as high demand is usually less frequent due to a sparser exercise routine 15.

There are two different mechanisms of stress fracture that should be distinguished to better understand the lesion pathophysiology and its consequences 7. These two types of injury are similar in results but opposite in their origin and are mainly differentiated by the previous state of the bony matrix. Insufficiency fractures occur when abnormal bone (osteopenic, low mineral density bone) is injured by a relatively normal load. In contrast, fatigue fractures occur when normal mineral density bone is repeatedly laden with abnormal high loads (overused) 8 and occurs through an sudden growth in frequency, duration, or intensity of activity when bone reabsorption (osteoclasts) oversteps its replacement (osteoblasts). As repetitive and cumulative loads are applied on the bone, it absorbs the forces by deforming its structure within the elastic range, maintaining the ability of returning to the original shape, until its yield point. When the stress surpasses the elastic range, microfractures form, and the bone acquires a persistent plastic deformity 7. T‐stress fractures occur when these microfractures aggregate into a bony cortical discontinuity 19, 20. In fact, bone histology affects not only the fracture mechanism but also its outcome; poorly perfused sites are subjected to delayed healing, risk of nonunion, and a prolonged recovery time to full activity 1. Risk factors can be categorized into extrinsic (intensity of stress related to the type of sport, the training regimen, the footwear used and the training surface 7, smoking 15, 21, 22, and alcohol consumption 15, 22) and intrinsic (gender, age, race, fitness level, biomechanical factors, and previous history of stress fracture) 23, 24 categories. There are reports of specific risk factors for female athletes: low body mass index (under 19 kg/m^2^), late menarche (over 15 years old), and participation in selected sports 2.

### Diagnosis

The diagnosis of stress fractures is not straightforward, as they are associated with an unspecific origin and clinical presentation. Early and accurate diagnosis is critical for effective management, the prevention of complications, and an early return to full activity 1. The most common presentation is pain related to physical activity that relieves with rest, accompanied by localized tenderness 7. Usually the first symptoms are related to a change in the intensity of normal activity of the athlete or the weekend warrior who, without regular training, irregularly performs vigorous activity 20. Clinical examination may find nothing more than local tenderness, which may or may not be associated with edema over the affected area 1. Normal cortical thickening, vascular arterial channel, Brodie's abscess, avascular necrosis, and various neoplasms are alternative diagnoses on conventional radiograph images 25.

### Imaging

When a stress fracture is suspected, the initial option for imaging is simply the radiograph 7. Although it is the first choice for diagnosis, this frequently gives a negative result, particularly in the early stages of injury when its sensitivity is at its lowest at approximately 10%. In later stages, one may identify periosteal thickening (normally perpendicular to a major trabeculae), focal endosteal or periosteal reaction, lucency, and identifiable fracture lines in one cortex with accompanying periosteal response 20. Although MRI is the most efficient diagnostic test (sensitivity 100%, specificity 85%), even in the early stages of the disease, it is usually the second choice method because it is more expensive and less widely available. It is used when there is a high clinical suspicion of a stress fracture, but plain radiographs are inconclusive. Computed tomography images may add diagnostic value, particularly in orthogonal fractures 7. Recently, the use of ultrasound as a diagnostic method for stress fractures has increased due to its efficiency and availability. Although limited to the assessment of superficial structures, it is capable of analyzing the surface of cortical bone, which reveals itself as an hyperechoic shell, and identifying areas of cortical thickening and hypoechoic callus 26. Bone scintigraphy (particularly three‐phase Technetium‐99 m‐methylene diphosphonate scans) is also used for the diagnosis of stress fractures. It has an excellent sensitivity for any metabolic bone activity imbalance, but its low specificity limits its accuracy in identifying these lesions; it is reported that approximately 40% of the injury locations identified in bone scintigraphy are asymptomatic sites 27. Diagnostic biopsy is not indicated in these cases as it does not provide useful information, and the presence of osteoblastic reparative callus can give stress fractures the appearance of an aggressive bone tumor. Furthermore, it can actually be deleterious as it further weakens the bone, possibly aggravating the stress fracture 20.

### Treatment and outcome

**Figure 5:**
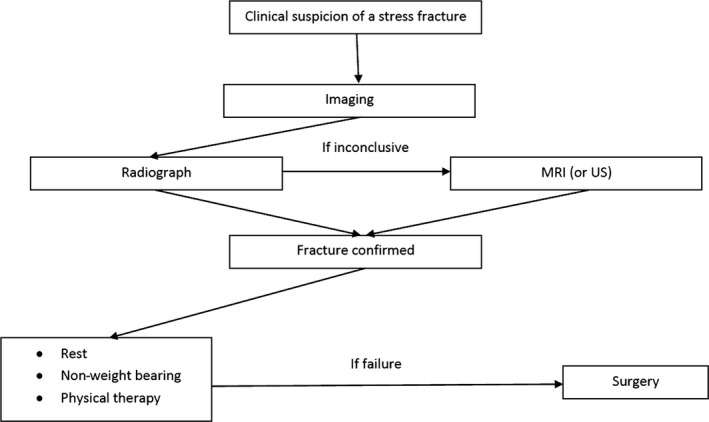
Recommended approach to lower limb stress fractures, in the older athlete.

Prevention is the first line of management for stress fractures; especially in female patients, the early screening of risk factors is a key point 2. Adequate physical activity during adolescence and encouraging participation in higher‐impact activities may promote general bone health 2. The equipment used should also be addressed in the prevention of stress injuries. It is generally suggested that training shoes be renewed every 6 months or every 500–800 km; especially in athletes with pes planus, the use of shock‐absorbent insoles and orthotics may reduce the risk of lower extremity stress fractures 28. Optimizing nutrition is also a key aspect. Although studies reveal mixed results involving vitamin D, its contribution to calcium and phosphorus metabolism seems to have an important role in stress fractures prevention and healing 29. Diet alone may not provide an adequate daily intake of vitamin D, so supplementation is often necessary^1^, with some reports pointing to its benefit in reducing the risk of stress fractures 16, 30. Generally, the treatment of a fatigue fracture is conservative–rest 31, limited weight bearing, and physical therapy. Surgical treatment may be indicated in some locations. Adequate differentiation must be made between low‐ and high‐risk stress fractures, and the management must be adjusted accordingly. The majority of cases are treated conservatively and resolve with a gradual return to previous activity levels and a low rate of complications (Fig. 5) 1.

## Conclusion

The incidence of stress injuries in older athletes is noticeably increasing, associated with a more active, older population. The key for the effective diagnosis of potential stress fractures is a careful review of the history of the patient and a clinical examination. Risk factors seem to be the same as for younger athletes, with the physiology of the injury believed to be multifactorial in both cases. The same principles apply for its prevention and treatment, but the implementation must be emphasized and overseen, as these older athletes usually present a more adverse outcome for the same lesion.

## Conflict of Interest

None declared.

## Authorship

All authors: contributed equally to the literature review and elaboration of this case report. AB, SK, and BR: compiled the original text and RG: revised the document and treated the patient presented in this case report.
